# War, emotions, mental health, and artificial intelligence

**DOI:** 10.3389/fpsyg.2024.1394045

**Published:** 2024-08-02

**Authors:** Kresimir Cosic, Vanja Kopilas, Tanja Jovanovic

**Affiliations:** ^1^Faculty of Electrical Engineering and Computing, University of Zagreb, Zagreb, Croatia; ^2^University of Zagreb Faculty of Croatian Studies, Zagreb, Croatia; ^3^Department of Psychiatry and Behavioral Neurosciences, Wayne State University School of Medicine, Detroit, MI, United States

**Keywords:** war, negative emotions, mental health and artificial intelligence, digital therapeutic devices, cognitive behavioral therapy, emotionally based strategic communications, ChatGPT and Gemini

## Abstract

During the war time dysregulation of negative emotions such as fear, anger, hatred, frustration, sadness, humiliation, and hopelessness can overrule normal societal values, culture, and endanger global peace and security, and mental health in affected societies. Therefore, it is understandable that the range and power of negative emotions may play important roles in consideration of human behavior in any armed conflict. The estimation and assessment of dominant negative emotions during war time are crucial but are challenged by the complexity of emotions’ neuro-psycho-physiology. Currently available natural language processing (NLP) tools have comprehensive computational methods to analyze and understand the emotional content of related textual data in war-inflicted societies. Innovative AI-driven technologies incorporating machine learning, neuro-linguistic programming, cloud infrastructure, and novel digital therapeutic tools and applications present an immense potential to enhance mental health care worldwide. This advancement could make mental health services more cost-effective and readily accessible. Due to the inadequate number of psychiatrists and limited psychiatric resources in coping with mental health consequences of war and traumas, new digital therapeutic wearable devices supported by AI tools and means might be promising approach in psychiatry of future. Transformation of negative dominant emotional maps might be undertaken by the simultaneous combination of online cognitive behavioral therapy (CBT) on individual level, as well as usage of emotionally based strategic communications (EBSC) on a public level. The proposed positive emotional transformation by means of CBT and EBSC may provide important leverage in efforts to protect mental health of civil population in war-inflicted societies. AI-based tools that can be applied in design of EBSC stimuli, like Open AI Chat GPT or Google Gemini may have great potential to significantly enhance emotionally based strategic communications by more comprehensive understanding of semantic and linguistic analysis of available text datasets of war-traumatized society. Human in the loop enhanced by Chat GPT and Gemini can aid in design and development of emotionally annotated messages that resonate among targeted population, amplifying the impact of strategic communications in shaping human dominant emotional maps into a more positive by CBT and EBCS.

## 1 Introduction

The impact of war and trauma on mental health is devastating, particularly for civilians who are living in a state of constant fear, hopelessness, misery, horror, sadness, and humiliation. Individuals in war-inflicted societies are subjected to profoundly traumatic and stressful events that can have detrimental effects on their mental health, leading to anxiety, depression, post-traumatic stress disorder (PTSD), and suicidal tendencies (Kleber, 2019; Rozanov et al., 2019; Jain et al., 2022). Prevalence rates of mental health disorders are strongly correlated with the number of traumatic events people have experienced during war time, as well as their individual stress resilience and vulnerability (Lim et al., 2022). A continuous flow of armed conflicts, global crises, natural disasters, and pandemics has resulted in an unprecedented number of individuals feeling stressed, anxious, depressed, and emotionally fragile (Jakovljevic et al., 2020; Ćosić et al., 2020a; Kopilaš et al., 2021; Lass-Hennemann et al., 2023; Mejia et al., 2023; Prazeres et al., 2023). Globally, yet unrecognized demand for high on-time effective preventive and treatment resources has contributed to a significant mental disease burden. This gap between what is needed and what is available has continued to grow due to the lack of mental health professionals and unmet need for early preventive treatment. Mental health disorders induced by war and trauma may lead to disorganization of key human emotional, cognitive and behavioral functions, like dysregulation of thoughts, emotions, feelings, compromised physiology, immune-inflammatory dysfunction, cognitive distortions and maladaptive coping mechanisms (Rozanov et al., 2019). The impact of war on the overall mental health and well-being might be catastrophic, surpassing the toll inflicted by any major disease in terms of mortality and disability. It can devastate entire nations, communities, families and individuals, frequently disrupting their socio-economic development and prosperity (Betancourt et al., 2018; Kleber, 2019; Rozanov et al., 2019; Trujillo et al., 2021; Lim et al., 2022).

The war-related death toll represents only the visible wounds of war, while numerous other consequences, such as the invisible wounds of war, like PTSD and suicides, are not yet efficiently and adequately addressed and treated. Potential of new mental health artificial intelligence (AI) tools and means might be effectively used in early recognition and treatment of mental health challenges. Therefore, this paper aims to address the capacity of a more comprehensive approach based on AI state-of-the-art tools and means to provide effective utility in coping with this global health challenge, particularly in societies affected by wars and traumas. Consequences of war, including forced displacement, exposure to violence, supplies’ deficiencies, damages to essential infrastructure, and disruption of vital services, can exert significant adverse effects on the psychological well-being and overall health of the Ukrainian population during and after the war (Hamama-Raz et al., 2022; Ellis et al., 2024). Measures of standard of living, psychological well-being, depressive symptoms, substance misuse, and consumption of unhealthy foods are commonly linked to the conflict regardless of sex, age, religion, or marital status (Konstantinov et al., 2023a,b). Our research highlights the importance of emotions as a motivating factor for engaging in voluntary service as well, since the majority of volunteers emphasized that feelings of compassion, anger, and a willingness influenced their decision to engage (Domaradzki et al., 2022).

## 2 The importance of emotion analysis

Global security challenges and wars might be expressed by different negative emotions, particularly those of fear, anger, despair, hatred, resentment, rage, and frustration (Milevski, 2020; Cricenti et al., 2022). These emotions may escalate into behavior of aggression, war, resistance, terrorism, insurgent action. Emotions may play a very important role in understanding human behavior and have a significant impact on the politico-security analysis. It is almost impossible to fully understand the complexity of global military tension, insecurity and armed conflicts without trying to understand the range and power of emotions in a theater of war. During the war, unlimited production and distribution of negative emotions makes multidimensional emotional space uncontrollable (Ćosić et al., 2012b). Therefore, the emotional dimension of any conflict or problem should not be underestimated. War tragedies and the negative emotion of humiliation generates irrational, harmful, devastating and hateful feelings. If emotions are not integrated and embedded into a global multidisciplinary politico-security analysis, the world will be in danger due to ignoring a fundamental aspect of human emotional behavior. Hence, strength and diversity of negative emotions remain a crucial factor in understanding the complexity of the global political and security landscape (Ćosić et al., 2018).

The explosion of negative emotions and their impact on relationship between nations, cultures, and religions is a crucial important topic which deserves much more attention. Without understanding the pivotal influence of emotions, which have greater control over individuals than they exert on them, it is fundamentally impossible to grasp the political and security realities of war trauma (Ćosić et al., 2012b; Webster and Albertson, 2022). For example, fear as absence of confidence can lead to obsessive worries about the present and the future and become more dangerous for overall security and individuals’ mental health. At the same time, fear is a force for survival in a dangerous environment and a natural protective response. Anger involves appraisals of relative strength and coping potential, while hatred is the strongest, extreme emotion which is characterized by willingness to harm and even annihilate the hated individual or hated group. However, war traumatic events can challenge and uproot related attachments, making their emotional nature exposed in a very visible manner (Bleiker and Hutchison, 2008).

In order to understand such complex societal situations, analysis of emotional contexts is extremely important and necessary (Ćosić et al., 2012b). Each emotion is related to a specific response tendency and action readiness (Frijda, 1987). This means that negative dominant emotional maps have corresponding action tendencies and behaviors, closely related to group willingness and energy to create some kind of change in the society.

An approach to use emotions as a mechanism to explain irrational behavior of war actors might be an innovative way to add value in searching for a solution for the most complex, unpredictable and uncertain war conflicts in the modern world. In order to reconcile people with diverse emotional landscapes, it is necessary to understand the main drivers of their behaviors and actions. Behavioral characteristics of people cannot be understood without deeper and complex analysis of their dominant emotional maps and their socio-cultural and security conditions and interactions. Different dominant emotions of the population must reflect in different policy ramifications (Mercer, 2005). This indicates that different societal and cultural values and norms cannot be easily changed by excessive use of military power. Finally, the effects of military dominance, like airstrikes or drone attacks, may produce strong negative emotions and effects in the battle for hearts and minds of a war inflicted population, making the use of military power a less effective (Dixon, 2009). Potential military failure might be a consequence of unrealistic expectations that serious security problems in war-inflicted societies can be resolved by the military while ignoring the fact that the post-conflict developmental and recovery programs are the most crucial (Galula, 1964).

## 3 Dominant emotional maps representation and mental health

The estimation and assessment of dominant emotions during and after war is extremely important, but at the same time a very challenging and complex task. In politico-security analyses the aggregation of dominant emotional maps, and their potential transformation into group actionable scenarios, actions, reactions, behavior, and riots, deserves more attention (Lofland, 1985). Participants in the same social situation often share a common awareness and a common emotional reaction to it (Barbalet, 1998; Pizarro et al., 2022). Aggregation and unification of these individuals’ emotional characteristics may lead to strongly unified emotional groups which may become powerful strategic agents in conflict management. Dominant emotional maps determine the ability of a society to cope with its own social and security challenges. Identifying the dominant emotions within populations in war-affected societies should be considered one of the foundational tasks. This endeavor aims to ameliorate negative emotions wherever they exist and capitalize on positive ones.

Group dominant emotional maps arise from the aggregation of emotional maps of individual members within a population (Ćosić et al., 2012a), and serve as representations of “dominant emotions,” which are publicly expressed feelings of collective behavior (Lofland, 1985). Additionally, they can be understood as depictions of “emotional atmospheres,” which encompass collective moods, or as “emotional climates,” which encompass collections of significant emotions or feelings that contribute to the formation and maintenance of political and social identities, as well as collective behavior (Barbalet, 1998; De Rivera et al., 2007). The synchronous alteration of dominant emotions among substantial portions of the population can potentially act as a driving force or leverage for broader societal transformations. The nuanced, but simultaneous modification of individuals’ feelings at the micro-level could lead to far-reaching transformations at the macro-level. Consequently, changes in dominant emotions entail shifts in the action tendencies of numerous individuals, thereby providing a basis for cohesive collective action on a societal scale.

**Figure 1:**
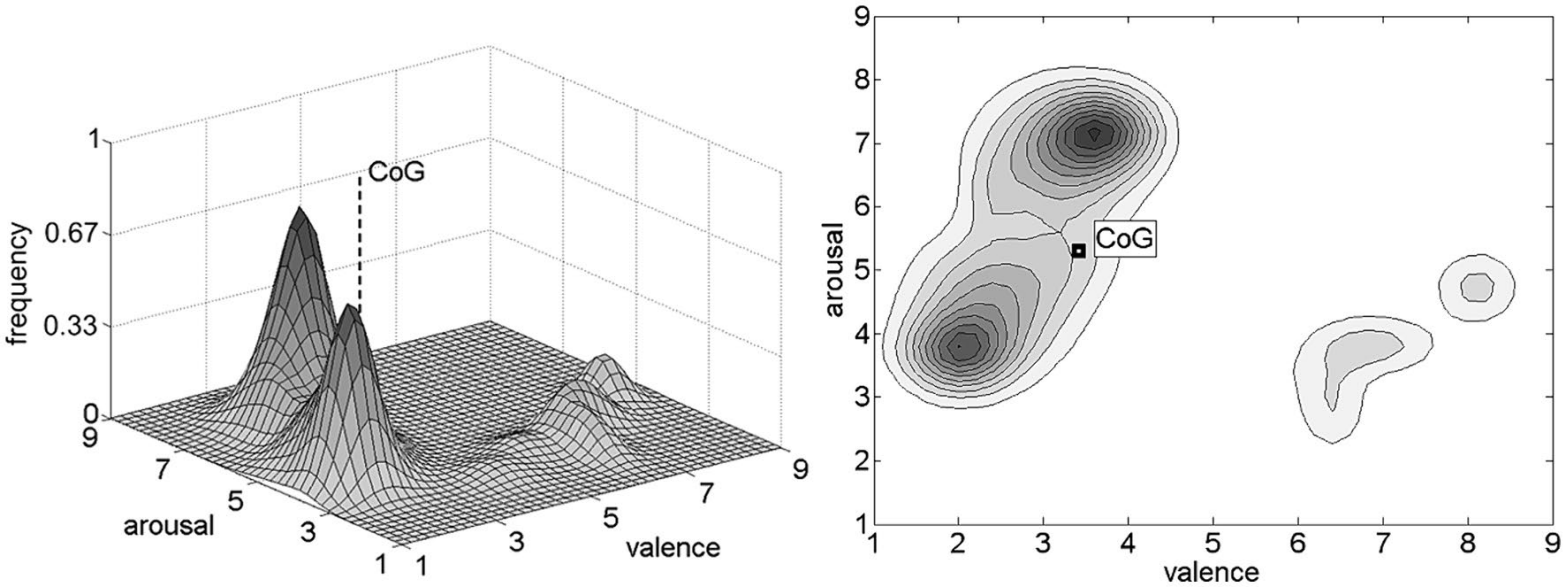
Hypothetical normalized emotional map of a war-inflicted population and the associated center of gravity (CoG): surface plot (left), contour plot (right).

Analyses of these dominant emotional maps in combination with other relevant efforts are the basis for a comprehensive approach to conflict resolution, and corresponding peaceful policy. Dominant negative emotions, particularly on the global and strategic political scene, are the main driving force of war and global insecurity (Webster and Albertson, 2022). Therefore, estimation and assessment of dominant negative emotion in times of war and trauma are extremely important. Figure 1 illustrates the hypothetical normalized negative emotional map during the war time. From the illustrated contour plots it is easy to notice that centers of gravity of dominant emotional maps are situated in the upper-left quadrant of the valence/arousal space, due to the presence of negative and mostly arousing emotions, like fear, anger, hatred, frustration etc.

Emotions affect attention, beliefs, and actions, focusing and guiding memory and influencing our cognitive processes (Tyng et al., 2017). They shape and strengthen our beliefs, help us rearrange our priorities and revise our goal hierarchies, influence our preferences, and act as a nexus among our beliefs, value systems and thereby strengthening our commitments (Gonzalez et al., 2020; Furman, 2024; Kisley et al., 2024).

The estimation of dominant emotional states may encompass various elements such as aggregated emotional feelings, beliefs, and a behavioral component characterized by impulsive or expressive gestures. Moreover, it incorporates a cognitive element associated with the evaluation of the scenario, as well as a drive component linked to the preparedness for activity (Scherer, 1984). As a result, when estimating dominant negative emotional maps, one must consider not only the collective emotional experience of a group, but also the corresponding behavioral and cognitive components (Ćosić et al., 2012a).

Dominant emotional maps can display data or signals from different sources, like linguistic textual records related to emotion from social media which are increasingly popular. Individuals experiencing mental health issues frequently express their psychological difficulties through various platforms and forums, such as Facebook, Instagram, Twitter, Reddit, and other online forums, by sharing text messages, comments, photos, videos, and other related information (Naslund et al., 2020; Zhang et al., 2022, 2023). A differentiating characteristic of Reddit, when compared to other sources of data, is the classification of posts into distinct subreddits based on specific subjects, such as anxiety, depression, post-traumatic stress disorder (PTSD), and even suicide (Zhang et al., 2022). In addition, electronic health records (EHR) function as a valuable source of secondary healthcare data, providing comprehensive documentation of individuals’ historical medical records. Another viable approach involves identifying mental illness by conducting user interviews and subsequently analyzing the linguistic information obtained from transcribed clinical interviews (Morales and Levitan, 2016; Spruit et al., 2022). Finally, the application of standardized questionnaires for individual’s diagnosis and self-assessment is also considered fitting in this circumstance (Ćosić et al., 2021).

Regarding mental health diagnostic analysis of keywords and statistical representations of words dominates, but involvement of state-of-the-art NLP methods, like large language models (LLM) can significantly improve word representations with better and deeper understanding of mental health context and semantics (Bartal et al., 2024). The interpretation of keyword-based features is much easier, while clarification of context and meaning of words is more abstract. Analyses of dominant emotional maps in war-inflicted societies reveal dominant emotions which people express in online posts or forums, such as fear, sadness and anger. Uncovering the overall emotional tone and how people describe their emotions in the war environment is the right approach to prediction and prevention of potential massive mental health diseases in a reliable and accurate way. Available NLP tools have a variety of computational methods to analyze and understand the emotional content of relevant textual data in war-inflicted societies, enabling better and deeper insights into their emotional states. From the utilization of NLP methodologies and illustrative large-scale data collections, we are able to observe intricate patterns of emotions and sentiments that are manifested in language and associated emotional and semantic characteristics that exhibit dynamic changes over time (Sawalha et al., 2022). Through the examination of posts, we can analyze linguistic features such as word frequencies, lexical diversity, narrative coherence, emotional content, and sentiment content to effectively detect and forecast significant mental health disorders (Ćosić et al., 2021). The most commonly used features in mental illness detection are linguistic patterns such as Bag-of-Words, Linguistic Inquiry and Word Count (LIWC), sentence and passage length, N-gram language models, emotional thesauri like WordNet-affect, and normative databases like the Affective Norm for English Words. Domain specific ontologies, dictionaries, and social attributes in social networks have the potential to improve accuracy. However, LIWC is the most widely used software tool in mental health research projects which is composed of more than 6,000 words, word stems and selected emotions and calculates approximately 90 output variables (Pennebaker et al., 2015).

## 4 Mental health challenges and potential of artificial intelligence

Disruptive AI-based technologies using machine learning (ML), deep learning (DL), neuro linguistic programming (NLP), cloud infrastructure, and new digital therapeutic apps offer a significant promise for enhancing mental health care on a worldwide scale, rendering these approaches more cost-effective and readily implementable, specifically targeting nations and individuals lacking sufficient access to health care (Schwalbe and Wahl, 2020; Lekkas and Jacobson, 2021; Koutsouleris et al., 2022; Tutun et al., 2023). Due to an inadequate number of psychiatrists and limited psychiatric resources to cope with war-related disasters, traumas and tragedies, new digital therapeutic wearable devices supported by advanced statistical methods and machine learning algorithms might represent a positive shift in the psychiatry of the future (Ćosić et al., 2021). The predictive AI-based methods which can recognize potential chronic psychopathology early enough compared to traditional reactive psychiatry is example of more proactive and preventive type of medicine (Ćosić et al., 2020a,b, 2021; Bertl et al., 2022). The major advantage of predictive AI is its ability to identify specific non-obvious patterns which are beyond human observation capabilities and may be essential for early detection of individuals at high risk of mental health deterioration (Ćosić et al., 2021). These AI-based tools and means are leading to a new era of mental health global management offering new opportunities and a more holistic approach to patients by early intervention with novel therapeutic methods (Schwalbe and Wahl, 2020; Garriga et al., 2022; Koutsouleris et al., 2022). These changes can be objectively assessed by state-of-the-art wearable devices which can capture clusters of different multimodal and multidisciplinary features focusing on early prediction and prevention of potential chronic mental health disorders using new AI-based therapeutic strategies, like computerized cognitive behavioral therapy (Wilhelm et al., 2020; Grodniewicz and Hohol, 2023; Khawaja and Bélisle-Pipon, 2023). Early prediction and prevention of mental health disorders, particularly for populations in war zones, based on the objective measurement and computation of relevant neuro-psycho-physiological and linguistic features and a machine learning model development of highly heterogeneous data sets, may enhance traditional face-to-face therapy. Such approach is particularly important for all of those who are exposed to high levels of stress during war time and need assistance more quickly and accurately.

Machine learning (ML) models have been developed to detect mental illness based on a combination of various multimodal physiological sensors (Chen et al., 2022; Garriga et al., 2022; Sabry et al., 2022). The computation of corresponding features, selection and labeling training datasets, and selection of validation and verification datasets using a supervised learning model, can be developed for classification or prediction of different mental health issues. Traditional and most common machine learning methods include: support vector machine (SVM), k-Nearest Neighbors (KNN), Adaptive Boosting (AdaBoost), Decision Tree (DT), Random Forest (RF), Naive Bayes (NB), and Logistic Regression (LR; Sarker, 2021). To develop a robust ML model, it is crucial to identify the primary multimodal features that serve as key predictors of severe mental health conditions (Zhang et al., 2022). The main advantage of supervised learning lies in the model***’***s ability to learn patterns from labeled datasets, therefore leading to better model performance. However, labeling the large amount of data with high quality is a time consuming and challenging job, therefore the methods that can help reduce the human annotation burden are of special interest. Methods which do not rely on labeled data or need only a small amount of data to train a classifier, are based on unsupervised learning methods which can discover patterns from unlabeled data, such as clustering data.

Currently, deep machine learning (DL) methods receive more attention and perform better than traditional machine learning methods, while the interpretable models, like XAI, deserve particular attention. DL methods can capture valuable features automatically without feature engineering and may be preferred for tasks of mental illness detection from text. DL methods consist of multiple layers, including an embedding layer and a classification layer (Zhang et al., 2022). The embedding layer has the ability to retain both semantic and syntactic information, thereby enhancing the training of deep learning models. Various embedding techniques exist, such as ELMo, GloVe word embedding, word2vec, and contextual language encoder representations like bidirectional encoder representations transformers (BERT). Depending on the structure of the classification layer, DL methods might be divided into convolutional neural networks (CNN), recurrent neural networks (RNN), transformer-based methods, and hybrid-based methods (Zhang et al., 2022). DL models have shown high accuracy in predicting mental health disorder diagnosis and severity (Garriga et al., 2022; Allesøe et al., 2023).

The utilization of NLP and the analysis of free-form online posts can provide significant advantages in accurately predicting potential psychiatric disorders (Le Glaz et al., 2021; Arowosegbe and Oyelade, 2023). In particular, distinguishing between individuals who are vulnerable to mental stress and those who are resilient to mental stress can greatly contribute to the reliability of such predictions (Ćosić et al., 2021). NLP-based semantic analysis and recognition of specific keywords to track individual emotions and moods illustrates promising improvements to empower mental health management (Zhang et al., 2022). Such linguistic features and information and related unstructured data may be valuable tool in identification of relevant keywords for early detection of various mental health problems (Ćosić et al., 2021). Early warning text-based indicators of potential mental illness deserve particular attention since they enable early intervention and prevention of serious mental health diseases, like PTSD. NLP methodologies possess the capability to apprehend irrational or distorted conversations that are related with some psychopathological patterns, such as the patient’s cognitive distortions, biases, core beliefs and negative wording. By employing NLP techniques, medical practitioners can unveil the configurations that exemplify how specific psychopathological conditions are manifested in language, as well as the corresponding semantic and acoustic qualities over a period of time (Ćosić et al., 2021). All individual posts, their contents, and comments on related, continuously monitored, social networks may serve as a source of relevant linguistic features such as word frequencies, lexical diversity, narrative coherence, sentiment of speech content and others, using them to classify major mental health disorders. These features might be calculated, for example by Linguistic Inquiry and Word Count (LIWC), Bag-of-Words model, word2vec and BERT.

Digital therapeutic devices and apps based on cognitive behavioral therapy and explainable artificial intelligence (XAI) might be an additional transformative and powerful approach in psychiatry of the 21st century leading toward highly efficient diagnosis and treatment as complementary assistance to traditional face-to-face therapy (Tong et al., 2022; Górriz et al., 2023). A virtual digital therapist might be as good as a human therapist based on the enormous potential of AI-based tools and means. Such applications offer great promise since they only require smart wearables sensors and smartphones. Cognitive behavioral therapy (CBT) is a type of evidence-based psychotherapy that is often recognized by the Institute of Medicine as the first-line approach to provide behavioral, cognitive, and emotional change and adaption to a range of common psychological and psychiatric problems (David et al., 2018). Finally, to the personalization of online CBT and XAI treatments using minimal human resources is an important research topic which deserves much more attention, particularly regarding the issues of optimal adaptive treatments, selection of best treatments, minimization of dropouts etc.

Explainable AI within the field of psychiatry could potentially serve as a self-explanatory digital aid to psychiatrists, enabling them to meticulously analyze vast amounts of data and identify intricate patterns and concealed indicators that may elude the perception of a psychotherapist. The goal of XAI in mental health diagnostics is to understand explanatory factors of mental illness in order to improve diagnostic performance and empower therapeutic decision-making (Kerz et al., 2023). The interpretability of diagnostics and treatments is extremely important for guiding psychiatrists to understand not only what has been extracted from big multimodal datasets, but also to enhance treatment and prediction (Koppe et al., 2021). Machine and deep learning-based methods achieve good performance by utilizing a large number of features, their extraction and computation, but they still fail to explain some complex decisions (Zhang et al., 2022). As a result, the explainability or interpretability of the machine and deep learning decision making process will become an important research direction in the future. This is an issue when both patients and doctors need to understand why an outcome has been produced for a given specific set of multimodal inputs. This allows physicians to check if the reasoning of the trained model aligns with their own understanding and general medical domain principles. Understanding the mechanisms and rationales behind a particular machine learning algorithm is crucial in establishing confidence and trust among domain experts. These factors can lead human experts to either reject or embrace these diagnostic approaches. Therefore, the need for transparency in psychiatry due to probabilistic variations relating to the complexity of neuro-psycho-physiological factors of mental health disorders is extremely important. As increasingly complex models are developed and their replacements for human expert decision making, it is necessary to ensure that the “black box” nature of these models have not learned undesirable patterns (Ali et al., 2023).

## 5 Mental health monitoring by wearable devices

Wearable and wireless sensors and sensing technology using a variety of AI algorithms can be applied in the prediction of different mental health disorders by monitoring human physiology, emotion, cognition, and behavior (Lee et al., 2021; Kalisperakis et al., 2023; Sigcha et al., 2023; Zheng et al., 2023). Majority of applications are deployed on smartphones or configured with a chatbot interface via a web application for providing access to a larger population offering a unique opportunity for ubiquitous mental health screening. Connected and embedded into people’s personal lives, smartphones provide considerable insight into individual routines, habits, activities, and human way of living. The convergence of sensor networks, fusion of sensors, processing of signals, and human interaction with these devices result in a vast amount of data, which serves as a valuable source for extracting intricate characteristics utilized by machine learning algorithms (Sabry et al., 2022). These algorithms are then able to identify and acquire meaningful patterns that aid in the prediction of potential mental health disorders. Wearable devices have the capacity to incorporate a range of sensor types, enabling the continuous monitoring of various bodily signals. Consequently, these devices can gather an extensive amount of data, potentially reaching millions of data points per person per day. Such information can be utilized by a range of machine learning techniques, like deep neural networks, for the purposes of training, learning, and predictive modeling (Ćosić et al., 2024). Smart wearables supported by AI can be very helpful in digital psychiatry to provide care to millions of potential patients simultaneously and can be used for clinical purposes to track patients with greater care and accuracy (Chen et al., 2022; Shajari et al., 2023). A large amount of the biometric data can be analyzed on edge computing devices enabling developers to secure sensitive data and enhance security and privacy. Based on innovation in new sensors technology and sophisticated algorithms, wearables can be used as diagnostic devices using machine learning models built on data streaming from each individual. It might be also very helpful to enhance traditional conventional face-to-face therapies which currently represent a serious barrier to care. These devices can resolve many problems regarding potential massive mental health problems induced by war traumas, natural disasters or pandemics.

Today, each part of human biology can be captured by more than a thousand sensors collecting a few thousand physiological, emotional, cognitive and behavioral features (Dang et al., 2023). Wearable physiological sensors may include measurements of heart rate variability, body temperature, breathing dynamics, oculometric features, electrodermal activity, respiration rate, blood volume, blood oxygen saturation, blood pressure, acoustic recordings, movement sensors, such as tri-axis accelerometers, tri-axis gyroscopes, inertial platforms, magnetometers, and many others (Ates et al., 2022; Scataglini et al., 2023). From all these neuro-psycho-physiological variables, more complex physiological states can be computed (i.e., respiratory sinus arrythmia). Daily tracking of activity and movement by GPS, their mutual interactions, and physiology or even metabolic functions, including variations of glucose, microbiome analysis, genomics sequencing, offers a new dimension in monitoring and tracking human behavior, and offers valuable tools and means in prevention of serious mental health disorders in war-inflicted societies.

Model development based on extracted features and sophisticated and complex innovative machine learning algorithms can be used for classification of certain mental disorders. For example, activity monitoring and recognition by text messages, location, movement, touch screen typing patterns, and voice recordings can assist in mental health monitoring and diagnostics. However, the interpretation of the impact of certain statistical features on classification or other outcome variables can often be challenging. Furthermore, the precision of a model can be adversely impacted by the incorporation of irrelevant characteristics (Sabry et al., 2022). It is important to note that the notion of “more is better” does not always hold true, as the inclusion of domain-specific features that possess expressive qualities tends to yield superior performance. For instance, features such as heart rate, breathing rate, changes in acceleration, motion jerk, and transient alterations in skin resistance can be considered as domain-specific features for seizure detection (Sabry et al., 2022). In certain applications, the focus lies on changes occurring over extended periods, while in others, attention is directed toward transient changes resulting from specific events like fall detection and emotion recognition. The growing array of wearable devices can lead to big changes in the prevention of chronic mental health diseases by continuous measurements to identify which patterns are normal and which are abnormal. Continuous measurements make it possible to establish which patterns are normal for a specific individual, such as respiration or heart rate, and can assist in recognizing important deviations before disease develops.

Acoustic characteristics of speech, like prosody have commonly been utilized as indicators of sentiments, emotions and underlying physiological states (Huang et al., 2021). Slight alterations in physiological and cognitive states can result in perceptible acoustic alterations in speech perception, particularly during distressing incidents (Scherer, 1984). Certain descriptions can be readily elucidated by individual characteristics such as reduced fundamental frequency of speech, decreased number of voiced frames in an utterance, and pauses between words. Additional set of acoustic features that delineate such phenomena contributes to more information in the emotional analysis of speech (Ćosić et al., 2021).

Challenges for machine learning applications on wearable devices are related to the accuracy of machine learning models. Cross-validation techniques increase accuracy by testing the model on unseen data that have not been used in training (Sabry et al., 2022). Model interpretability is crucial, especially in wearable device applications in healthcare, where users need understandable results. Model size also matters for wearable devices due to memory constraints, as does computational complexity for inference and online training. Past obstacles in model development include data collection, feature selection, and model evaluation, highlighting the need for caution in relying on machine learning decisions. Healthcare models must generalize well, handle unseen examples, consider personal attributes, offer interpretable results, and communicate outcomes carefully (Sabry et al., 2022).

## 6 Transformation of dominant emotional maps

Transformation of dominant emotional maps might be undertaken by simultaneous combination of computerized CBT (CCBT) on an individual level, as well as usage of emotionally based strategic communications (EBSC) on a public level. Strategic communications, aimed at shaping perceptions and behavior, can be enhanced by leveraging emotions, as proposed in EBSC (Ćosić et al., 2012a,b). This aligns with J. A. Treadwell’s statement: “If you want to influence someone, you have to touch their emotions” (Merle, 2005). Emotionally infused strategies, whether in individual therapy like CCBT or in strategic communications like EBSC, are essential for effective intervention. Integrating emotional strategies into strategic communications can be pivotal in conflict and post-conflict management within diverse social and cultural contexts. Hence, the proposed positive emotional transformation by means of CBT and EBSC may provide important leverage in efforts to protect mental health of local populations in war-inflicted societies. The transformation of negative dominant emotional maps into more positive emotions delivered by EBSC messages to targeted audiences is focused on mental health protection and support. This type of psychological operation can be defined as “planned psychological activities using methods of communications and other means directed to selected audiences in order to shape their perceptions, attitudes and behavior to achieve specific political and military objectives” (Reding et al., 2010). Strategic communication can be defined as “a systematic series of sustained and coherent activities, conducted across strategic, operational and tactical levels to promote and sustain particular types of ideas, opinions and behavior” (Tatham, 2008).

**Figure 2:**
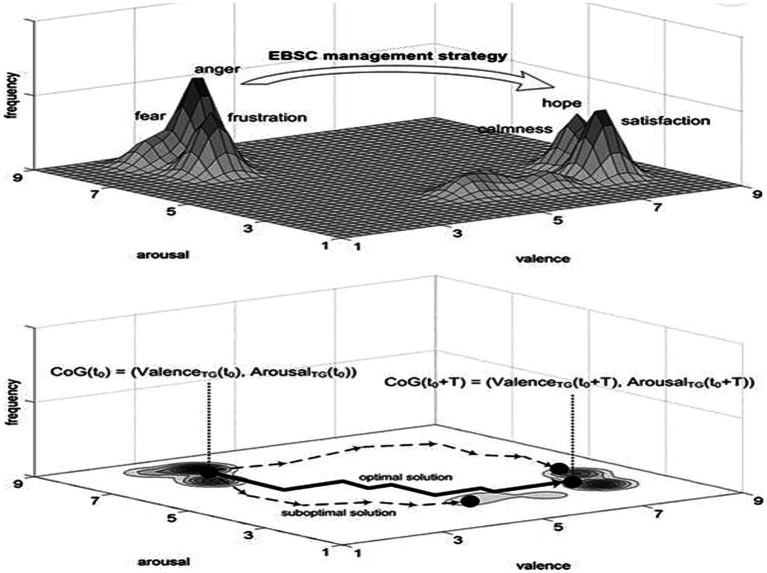
Transformation of a negative dominant emotional map of a war-inflicted society to a more positive dominant emotional map by EBSC.

The idea of EBSC originates from our research focused on new technologies in digital psychiatry, like Virtual Reality (VR) adaptive stimulation (Ćosić et al., 2010). The similarity between individual psychotherapy, such as Virtual Reality Exposure Therapy (VRET) and their generalization to psychological operations on a strategic level, based on EBSC, is based on their common neurobiological background in “emotional brain” (Wiederhold and Wiederhold, 2008; LeDoux, 2012; Ćosić et al., 2012a). The best information contents, context, and emotional properties delivered by EBSC are related to ideas and emotions that quickly resonate among targeted audiences and cause a small incremental positive emotional step toward desirable dominant emotional maps (Ćosić et al., 2018). In other words, EBSC must be delivered as a comprehensive communication strategy through which individual and group emotions are reshaped in a more positive manner, producing and shaping “soft power” important for reaching the desired emotional end state (Ćosić et al., 2012a). Figure 2 illustrates the transformation of a negative dominant emotional map of a war-inflicted society to a desirable more positive dominant emotional map, which could be facilitated by EBSC. Redirection of attention of more vulnerable individuals or selected groups from dominant negative emotions to more positive emotions may change how people appraise their current war situation, stimulating incremental emotional transformation toward more positive emotional and mental states.

AI-based tools that can be applied in the creation of EBSC stimuli, like Chat GPT or Google Gemini (Alford, 2024), may have great potential to significantly enhance EBSC by deeper understanding of the dominant emotional maps in targeted populations by more comprehensive semantic and linguistic analyses of available text datasets (Elyoseph et al., 2023). Chat GPT and Gemini can aid in crafting emotional messages that resonate among the targeted populations, amplifying the impact of strategic communications and reshaping dominant emotional maps over time into more positive ones, simultaneously protecting human mental health in war torn societies. It is important to stress that Gemini has an advantage over Chat GPT in EBSC providing high-quality information extracted in real-time, having been trained from Gmail, Google Docs, Google Maps, and Google Drive accounts instead of books and articles to ensure more up-to-date information. However, before one can fully rely on these AI tools, the applied LLM must provide high accuracy, transparency, and trust. Off-the-shelf models such as ChatGPT 4 or Gemini can produce wrong answers which is unacceptable in any kind of public campaigning, therefore human experts must be permanently embedded in the EBSC closed loop. To be applicable, understandable, and explainable within a specific war zone or region, LLM should be trained on a relevant dataset, for example, using Ukraine or Gaza posts related to current security environment and war disasters.

Training Chat GPT for such a specific strategic communication issue requires fine-tuning the model on a dataset relevant for the selected use cases. The collection of war domain-specific datasets from different local data sources should reflect the emotional and mental states covering a wide range of scenarios and questions that users might encounter within the selected use case domain. After cleaning and preprocessing the collected data to remove noise and tokenization, the preprocessed dataset is used to fine-tune a pre-trained ChatGPT model. Techniques like transfer learning might be used to leverage the knowledge already encoded in the pre-trained model. Training the fine-tuned model on the selected use case specific dataset is monitored by tracking metrics such as loss and validation performance. Once training is completed, evaluation of the performance of the fine-tuned model using validation data, or by manually testing it with sample inputs from related domain. Once the model has satisfied required performance metrics, it can be deployed in an EBSC campaigning process. Continuous monitoring of its performance in production requires its fine-tuning based on user feedback and real-world usage. Real time monitoring and tracking of emotional dynamics based on different data sources with a variety of NLP tools and methods within specific war zone and consequently mental health states deteriorations is extremely important. Early detection and prevention of serious mental health disorders just in time and online comprehensive state of the art therapy is prerequisite for meaningful success.

## 7 Conclusion

The power of simultaneous usage of CCBT and EBSC supported by tools and means of AI may provide a breakthrough in global mental health recovery in war-inflicted societies after the enormous psychological distress caused by war brutality and tragedies. The synergy between individual psycho-therapeutic techniques, such as Virtual Reality Exposure Therapy (VRET), and psychological operations on a strategic level, based on EBSC can be understood by their common neurobiological underpinnings (Wiederhold and Wiederhold, 2008; Ćosić et al., 2012a,b). The potential of AI-based tools and means, such as machine learning, deep learning, neuro-linguistic programming, cloud infrastructure, and new wearable therapeutic devices and apps offer an immense prospect to enhance mental health care on a global scale, rendering them more economically viable and readily implementable. Specifically, when considering the insufficiency of psychiatrists and the limited availability of psychiatric resources in addressing war traumas, disasters, and tragedies, the suggested approach may prove to be a transformative force in the field of psychiatry in the years to come.

Emotionally based strategic communications supported by ChatGPT and Gemini have the potential to revolutionize digital psychiatry in combination with previously described tools and means based on multimodal physiological features, wearable and wireless devices, machine learning and edge and cloud computing. Sustainability of any long-term solution in war zones will depend on the proposed approach, bringing the new vision on how to cope with and overcome the invisible wounds of war trauma.

## Author contributions

KC: Writing – review & editing, Funding acquisition, Resources, Supervision, Writing – original draft. VK: Writing – review & editing, Resources. TJ: Supervision, Writing – review & editing.
